# Primary pancreatic lymphoma – pancreatic tumours that are potentially curable without resection, a retrospective review of four cases

**DOI:** 10.1186/1471-2407-6-117

**Published:** 2006-05-04

**Authors:** Peter S Grimison, Melvin T Chin, Michelle L Harrison, David Goldstein

**Affiliations:** 1 Institute of Oncology, Prince of Wales Hospital, Sydney, Australia; 2 Faculty of Medicine, University of New South Wales, Sydney, Australia

## Abstract

**Background:**

Primary pancreatic lymphomas (PPL) are rare tumours of the pancreas. Symptoms, imaging and tumour markers can mimic pancreatic adenocarcinoma, but they are much more amenable to treatment. Treatment for PPL remains controversial, particularly the role of surgical resection.

**Methods:**

Four cases of primary pancreatic lymphoma were identified at Prince of Wales Hospital, Sydney, Australia. A literature review of cases of PPL reported between 1985 and 2005 was conducted, and outcomes were contrasted.

**Results:**

All four patients presented with upper abdominal symptoms associated with weight loss. One case was diagnosed without surgery. No patients underwent pancreatectomy. All patients were treated with chemotherapy and radiotherapy, and two of four patients received rituximab. One patient died at 32 months. Three patients are disease free at 15, 25 and 64 months, one after successful retreatment. Literature review identified a further 103 patients in 11 case series. Outcomes in our series and other series of chemotherapy and radiotherapy compared favourably to surgical series.

**Conclusion:**

Biopsy of all pancreatic masses is essential, to exclude potentially curable conditions such as PPL, and can be performed without laparotomy. Combined multimodality treatment, utilising chemotherapy and radiotherapy, without surgical resection is advocated but a cooperative prospective study would lead to further improvement in treatment outcomes.

## Background

Primary pancreatic lymphoma (PPL) is rare, comprising less than 0.5% of pancreatic tumours[1]. To distinguish PPL from secondary involvement of the pancreas by non-Hodgkin's lymphoma, Behrns' clinical and diagnostic criteria of PPL include: mass predominantly within the pancreas with grossly involved lymph nodes confined to the peripancreatic region, no palpable superficial lymphadenopathy, no hepatic or splenic involvement, no mediastinal nodal enlargement on chest radiograph, and normal white cell count [2]. Presenting symptoms are non-specific, typically including abdominal pain, weight loss, nausea and vomiting [2]; but also jaundice, acute pancreatitis, and small bowel obstruction [3]. PPL can be difficult to differentiate from pancreatic adenocarcinoma without definitive pathological diagnosis[3], and correct diagnosis is crucial given that PPL has differing management and usually a much better prognosis. The sizeable cohort of patients with PPL who are not cured with current treatment demands further improvements. Optimal treatment of PPL remains controversial, particularly the role of surgery and radiotherapy.

A retrospective analysis of patients with PPL at our institution was conducted, to examine our treatment outcomes with modern multimodality therapy. We present a case series of four patients – one of the few in the recent literature when newer therapies such as rituximab have become available – and contrast outcomes with previously published case series.

## Methods

An electronic search through the medical oncology department records of Prince of Wales Hospital (Sydney, Australia) identified four cases of PPL among 481 cases of non-Hodgkin's lymphoma between 1990 and 2005. Medical records were reviewed, in regards to age, sex, presenting symptoms, radiological appearance, histological diagnosis and method, staging investigations, treatment regimen and outcome.

Staging was assigned by a modification of the Ann Arbor classification [4]; accordingly stage IE disease is confined to the pancreas, and stage IIE disease involves the pancreas and peripancreatic nodes. Histological categorisation was according to the Revised European American Lymphoma (REAL) classification [5]. Performance status was defined according to the Eastern Cooperative Oncology group (ECOG) scale [6]. The prognosis of patients was assessed using the criteria of the International Prognostic Index [7] for aggressive lymphomas, and the Follicular Lymphoma International Prognostic Index (FLIPI) [8] for follicular lymphomas respectively.

Previously reported cases of PPL published between 1985 and 2005 were identified through a Medline search of the English literature using the keywords of "pancreas" and "lymphoma". Further case series were identified through citation review of identified articles. Single case reports, and cases of stage III/IV disease were excluded.

## Results

### Case series

Four cases were identified of this rare condition at our institution. All cases were male, and age ranged from 56 to 70. Summary data is presented in table 1.

### Case 1

**Figure 1:**
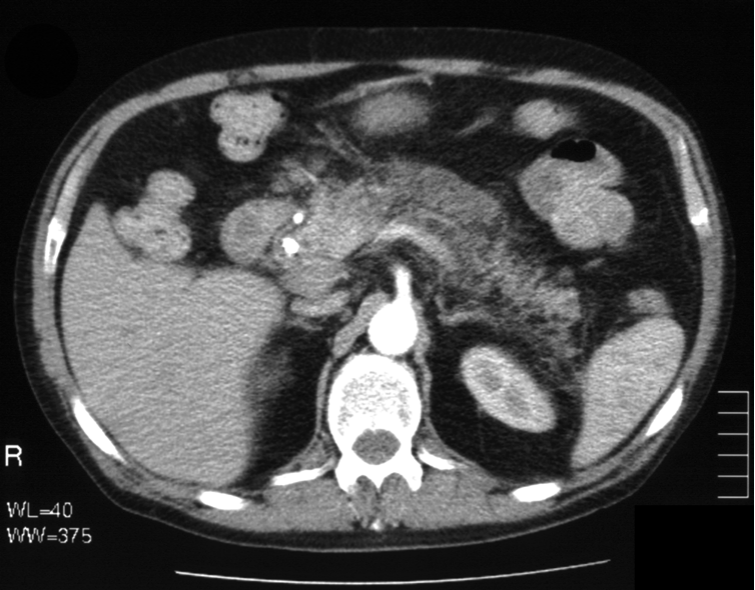
**Case 1 CT Scan**. CT scan of upper abdomen in arterial phase shows low density soft tissue in region of pancreatic head and posterior to body of pancreas, and biliary stent

A 64 year old man presented with a five week history of right upper quadrant pain, nausea, and jaundice, with five kilograms of weight loss. Liver function tests demonstrated biliary obstruction. Serum Ca-19.9 tumour marker assay was markedly increased at 500 kU/L (normal range 0–40). Abdominal CT demonstrated a 6 cm solid mass within the head of the pancreas, invading the portal vein, associated with biliary obstruction, but without lymphadenopathy or hepatic involvement (Figure 1). At ERCP a long lower common bile duct stricture was stented with prompt relief of biliary obstruction. Although pancreatic adenocarcinoma was strongly suspected, endoscopic ultrasound-guided biopsy revealed CD20 positive diffuse large B cell non-Hodgkin's lymphoma. Staging PET scan and bone marrow aspirate and trephine (BMAT) noted uptake in the coeliac nodes, without evidence of widespread dissemination of lymphoma.

Pre-operative assessment avoided the need for exploratory laparotomy. 4 cycles of CHOP (Cyclophosphamide, Doxorubicin, Vincritine, Prednisone) with concurrent rituximab (anti-CD20 chimeric monoclonal antibody, Genentech, CA, USA) was administered with complete response. Chemotherapy was then ceased because of asymptomatic anthracycline-induced decline in ejection fraction. Involved field external beam radiotherapy (36 Gray in 20 fractions) was administered. Fifteen months following diagnosis the patient remains in complete remission.

### Case 2

**Figure 2:**
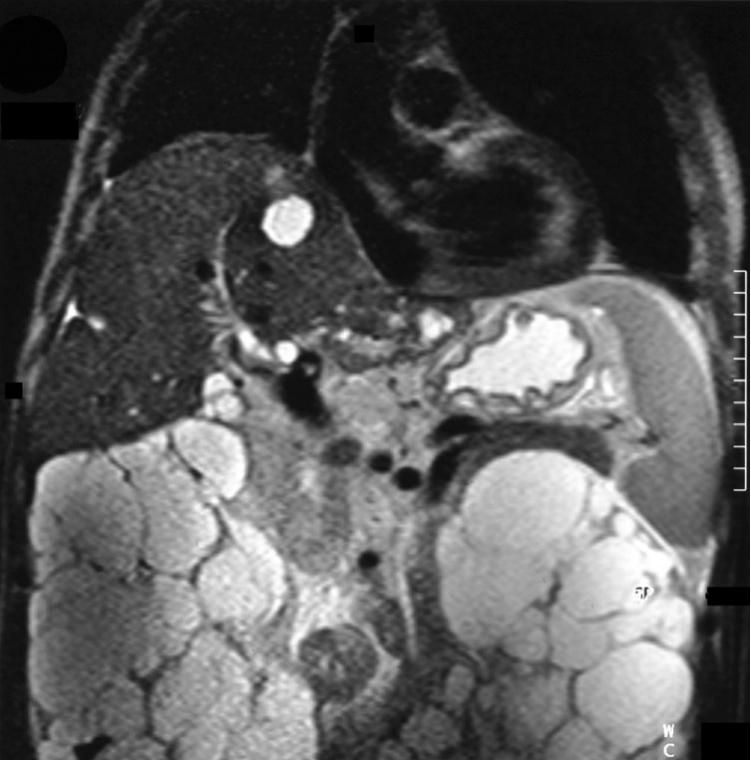
**Case 2 MRI**. MRI (magnetic resonance imaging) shows mass arising from head of pancreas, as well as hepatic and renal cysts.

**Figure 3:**
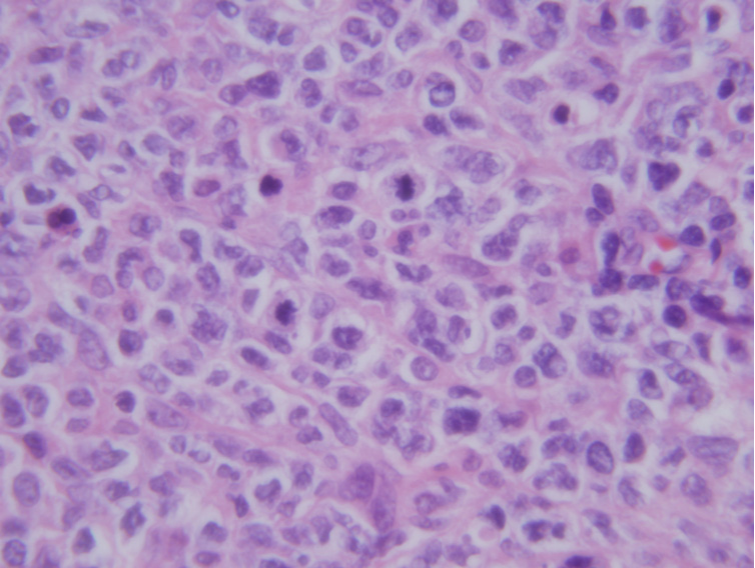
**Case 2 Biopsy**. Histological section shows large mononuclear cells, consistent with diffuse large B-cell lymphoma (H&E stain, ×200)

**Figure 4:**
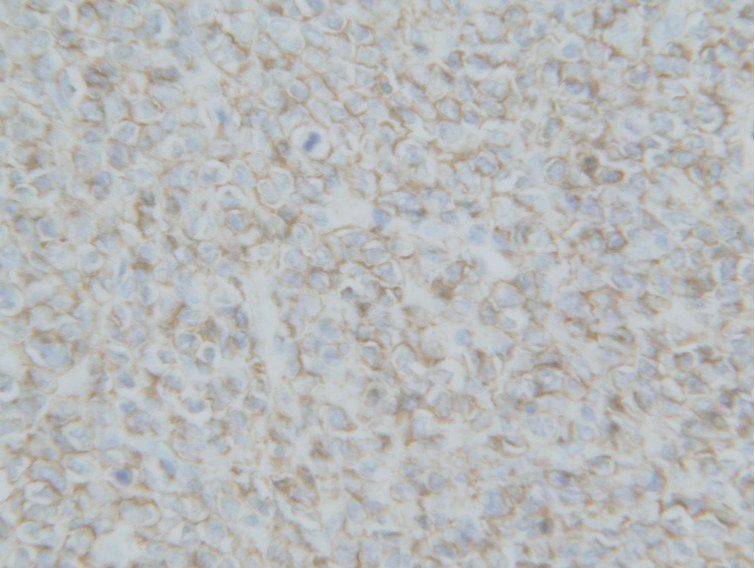
**Case 2 Biopsy CD20 stain**. CD20 positive staining of cells, consistent with B cell origin (×200)

A 70 year old man described a several month history of intermittent vomiting, anorexia and weight loss, on a background of chronic renal failure due to polycystic kidney disease (serum creatinine 450 umol/L; normal range 60–110 umol/L). MRI (magnetic resonance imaging) of the abdomen (Figure 2) demonstrated a large confluent mass lying above the head of the pancreas, posterior to the stomach, as well as prominent renal and hepatic cysts, without lymphadenopathy or mesenteric vessel involvement. Ca-19.9 was mildly elevated. CT-guided FNA was non-diagnostic, and endoscopic ultrasound was not available. At surgery – a risky procedure given his renal impairment – a large tumour was noted posterior to the duodenum, with bulky lymphadenopathy and peritoneal seeding. Although pancreatic carcinoma or polycystic kidney disease of the pancreas was suspected, intra-operative frozen section revealed CD20 positive diffuse large B-cell lymphoma (Figure 3 and 4). Formal staging with gallium scan and BMAT did not reveal further dissemination. Given potential for cure with chemotherapy and radiotherapy, resection was not performed.

Systemic combination chemotherapy with dose-modified CHOP was administered, after explanation of risk of significant toxicity and treatment-related end-stage renal failure. Dose-limiting toxicity included neutropaenia, cardiotoxicity and sensory neuropathy, which required cessation of treatment after only three of a planned six cycles. Progress CT scan demonstrated over 50% reduction in size of the pancreatic mass. Involved field radiotherapy (45 Gray in 25 fractions) was well tolerated, inducing radiological complete remission.

Thirty months after diagnosis end-stage renal failure occurred, requiring hemodialysis. Thirty-two months following diagnosis (23 months following therapy) the patient suffered symptomatic mediastinal and gastric recurrence. Second-line combination chemotherapy (DHAC – carboplatin, doxorubicin, cytosine arabinoside, dexamethasone) was poorly tolerated, with febrile neutropaenia and grade three gastrointestinal bleeding. The patient had a limited response, and died a short time later.

### Case 3

A 56 year old man presented with progressive dyspepsia and 12 kg of weight loss over 18 months. Ultrasound and CT revealed a five cm pancreatic mass involving the transverse colon; without lymphadenopathy, mesenteric vessel or hepatic involvement. At laparotomy for a planned Whipple's procedure, histopathology from open biopsy of the pancreatic mass and adjacent nodes was consistent with CD20-positive grade II (mixed small and large cell) follicular lymphoma. PET scan and bone marrow staging revealed regional nodal involvement. Resection was not performed.

Treatment consisted of involved field radiotherapy (36 Gy in 20 fractions), with sequential CVP chemotherapy for five cycles and sequential rituximab for four cycles. Restaging confirmed complete response, and the patient remains in complete remission 25 months following diagnosis.

### Case 4

A 61 year old man experienced progressive epigastric and back pain, 12 kg weight loss and then jaundice. Liver function tests were consistent with biliary obstruction. Ca19.9 was within normal range. CT scan revealed a pancreatic mass and peripancreatic lymphadenaopthy, without mesenteric vessel or hepatic involvement. ERCP and insertion of biliary stent relieved obstruction, however biliary brushings and subsequent FNA were non-diagnostic. Laparotomy and open biopsy of the peripancreatic lymph nodes revealed CD20 positive diffuse large B-cell lymphoma, and resection was not performed.

Treatment consisted of three cycles of CHOP chemotherapy followed by involved field radiotherapy (42 Gy in 19 fractions). The patient had a complete response to treatment, however relapsed out of field 21 months following diagnosis (17 months after treatment), with complete response to second-line ICE chemotherapy, high-dose BEAM chemotherapy and autologous stem cell transplantation. After 64 months following diagnosis (37 months from 2^nd ^treatment), the patient remains in remission.

### Literature review

Literature review identified 103 additional cases of PPL from 11 case series. Histopathology; use of successful non-operative biopsy; use of resection, chemotherapy and radiation; and outcomes are described in table 2.

High-grade B cell lymphomas were most commonly identified (45% of cases), followed by low grade B lymphomas (15%) and other B cell lymphomas (34%). T cell lymphomas, although very uncommon (4% of cases), carried a dismal prognosis. Our series was comparable to other series with 75% high-grade B cell lymphomas and 5% low grade lymphomas. Histological diagnosis was established by non-operative biopsy in 29 of 105 cases (28%), laparotomy in 69 of 105 cases (65%), and autopsy in 7 of 105 cases (7%). Surgical resection of the tumour was performed in 23 of 107 cases (21%). Chemotherapy was administered in 80 of 107 cases (75%). Radiotherapy was administered in 33 of 105 cases (31%).

Comparisons were made between treatment groups. Without any definitive treatment, results were uniformly poor with no patients free of disease and poor long-tem survival. Patients treated with chemotherapy and/or radiotherapy – without resection – did not appear to have worse outcomes than resected patients. Formal statistical comparison was not able to be performed.

In our series of non-surgical multimodality therapy, three of four patients were free of disease at time of follow up, with mean survival 34 months, and 2-year survival of 75%.

## Discussion

Our case series illustrates that the clinical presentation of PPL can be difficult to differentiate from pancreatic adenocarcinoma without definitive pathological diagnosis. Reliance on symptoms, imaging and tumour markers – in the absence of definitive pathological diagnosis of suspected pancreatic adenocarcinoma – can potentially result in the misdiagnosis of a small minority of potentially curable patients. This is important as the prognosis and management of PPL differs greatly from that of adenocarcinoma.

Appearances on CT can be helpful to differentiate the two conditions, but are not definitive[9,10]. Ca19-9 is the most useful tumour marker in pancreatic carcinoma, but can be misleading as it may also be elevated in other malignancies, particularly of the upper gastrointestinal tract, including PPL as described in case one [11]. Without definitive pathologic diagnosis, potentially curable conditions such as PPL; as well as other malignancies with more favourable prognosis, including periampullary, distal common bile duct, duodenal and mucinous cyst adenocarcinomas, may be misdiagnosed[12].

Non-operative evaluation and biopsy of pancreatic masses can avoid the need for invasive surgery, if conditions such as PPL are found. The majority of cases of PPL in our literature review required laparotomy for diagnosis, which may have been avoided with successful non-operative biopsy and modern combined modality treatment. Radiological-guided percutaneous FNA of the pancreas is a very useful technique, which requires experienced radiologists and cytopathologists to obtain a diagnosis on a small amount of tissue[3]. Endoscopic ultrasound has greatly improved the accuracy of diagnosis and obtaining diagnostic tissue [13-18]. Diagnosis of PPL may be extremely difficult on haematoxylin-eosin stains alone, and resemble poorly differentiated carcinoma and reticulum cell sarcoma[19], thus immuno-histochemical stains and flow cytometry are essential[3]. It must be emphasised that cytological diagnosis may not be adequate for diagnosis and categorisation of an abdominal mass, and tissue biopsy should be considered. In some situations (including two of our patients with non-diagnostic FNA), laparotomy may be required for definitive diagnosis.

Treatment of PPL remains controversial – particularly the role of surgery and radiotherapy – and based on our findings we do not support routine pancreatectomy. All patients in our case series received multimodality treatment with both chemotherapy and radiotherapy, without surgical resection. Experience from our case series and literature review (table 2) indicates that this modern treatment regime achieves favourable outcomes, comparable or better than surgical series, without the morbidity of surgical resection. Most modern authors would not recommend surgery except when non-surgical diagnosis is unsuccessful [20-22]. Surgery is difficult in PPL because tumours are large, and often associated with an otherwise histologically normal pancreas, carrying a high risk of postoperative pancreatic fistula[23]. Technical improvements in pancreatic surgery have led to reduced peri-operative morbidity and mortality, and in contrast Koniaris argues that pancreatectomy should be reevaluated as a method of improving local control and cure rates[23].

It is pertinent to consider the diminishing role of surgery in other localised extra-nodal lymphoma. Historically, the treatment of localised gastric non-Hodgkin's lymphoma, excluding MALT (mucosa-associated lymphoid tissue)-type lymphomas, was based on surgery – to ensure adequate diagnosis and staging, and maximise survival rates [24]. Three recent large prospective studies [24-26] and two smaller randomised controlled trials [27,28] of chemotherapy vs. combined surgery and chemotherapy have reported equivalent survival. Thus the role of surgery in gastric lymphoma may be limited to rare patients with acute complications or residual disease following non-surgical treatment [26,29] In intestinal non-Hodgkin's lymphoma, following a prospective study there is a trend away from extensive resection [30]. Localised extraintenstinal non-Hodgkins lymphoma has been treated without surgery for over two decades, following numerous well conducted prospective clinical trials. These changes in management illustrate the need for large cooperative prospective studies in PPL, which will better define any benefit of resection, as well as the role of therapies such as radiotherapy and rituximab.

Radiotherapy has had only limited reported use in the treatment of PPL (see table 2). Potential concerns about the safety of radiotherapy in this region may be unnecessary, as toxicity has been substantially reduced with three-dimensional treatment planning and conformal delivery of radiotherapy[31]. With these modern techniques small bowel toxicity is minimised, and none of our patients required radiotherapy interruption. The role of radiotherapy in early stage high-grade lymphoma remains controversial in a global sense. In localised intermediate and high-grade non-Hodgkin's lymphoma, chemotherapy using the CHOP regimen plus adjuvant radiotherapy is superior to chemotherapy alone[32]. Intensive chemotherapy regimens without radiotherapy have been shown to be superior to CHOP plus involved field radiotherapy[33]. An important question asks whether the addition of adjuvant radiotherapy to more intensive chemotherapy regimens would further improve outcomes[34]. We advocate combined multimodality therapy with both chemotherapy and radiotherapy for PPL.

Two of our four patients received rituximab (anti-CD20 chimeric monoclonal antibody, Genentech, CA, USA). We are not aware of its use in other series of PPL, however its use has been reported in gastric lymphoma [35], and evidence exists of its benefit combined with chemotherapy in other high-grade lymphoma for patients aged over 60[36].

Biliary sepsis is a potential problem of multimodality therapy in the setting of PPL, due to both the frequent presence of biliary stents, and the risk of neutropaenia. A study of endobiliary stents in multimodality therapy of pancreatic carcinoma reported low rates of complications, with 15 of 101 cases complicated by occlusion or migration, and no uncontrolled biliary sepsis or stent-related death[37]. Another study of patients with malignant biliary obstruction and bile duct stents did not find increased biliary complications in patients receiving chemotherapy[38]. Metal stents are superior to plastic stents for long-term patency [39-41]. Prompt recognition and treatment of biliary complications is important to allow ongoing therapy, as happened on several occasions to our patients.

We have examined for any possible association between polycystic kidney disease and PPL, given the existence of both conditions in case two. Although polycystic kidney disease has been associated with pancreatic cystadenoma and cystadenocarcinoma in a small number of case reports [42-44], we are not aware of any association with pancreatic lymphoma.

The study of PPL is limited by the rarity of the condition, and the consequent lack of randomised trials or large case series. The comparison of our case series with other series must be interpreted cautiously, given variable follow-up (range two – 108 months), incomplete data in some case series[2,3,12,45], and the inherent publication bias within case series favouring positive results, as described by Albrecht[46]. We strongly advocate a multi-centre prospective study of patients with PPL to improve patient outcomes.

## Conclusion

PPL is a rare but potentially curable pancreatic tumour, and mandates pathological diagnosis of all pancreatic masses, as its treatment and prognosis differs from adenocarcinoma. Non-operative diagnosis may avoid the need for surgery, as outcomes with chemotherapy and radiotherapy – without surgical resection – compare favourably to surgical series. Nevertheless a sizeable cohort of patients are not cured with modern therapy, and we advocate a prospective study to further improve treatment outcomes.

## Competing interests

The author(s) declare that they have no competing interests.

## Authors' contributions

PSG collected the data for the case series and the literature review, and drafted the manuscript. MC also collected data for the case series. MH also drafted the manuscript. DG conceived the study, participated in its design and coordination, and helped to draft the manuscript. All authors read and approved the final manuscript.

## Pre-publication history

The pre-publication history for this paper can be accessed here:



## Figures and Tables

**Table 1:** Patient characteristics, treatment and outcomes

	Case 1	Case 2	Case 3	Case 4
Age	64	70	56	61
Sex	Male	Male	Male	Male
Presenting Symptoms	Abdominal pain, nausea, weight loss then jaundice	Vomiting, weight loss	Dyspepsia, weight loss	Abdominal pain, weight loss then jaundice
Delay until diagnosis	5 weeks	3 months	18 months	4 months
Method of diagnosis	Endoscopic ultrasound FNA	Laparotomy	Laparotomy	Laparotomy
CT appearance	6 cm pancreatic mass with biliary obstruction and portal vein involvement	Large peri-pancreatic mass	5 cm pancreatic mass	Pancreatic mass and peripancreatic lymph nodes
Ca-19.9 level*	500 kU/L	50 kU/L	Not available	39 kU/L
Pre-treatment LDH	Normal	Elevated	Normal	Elevated
Histology**	DLBCL	DLBCL	Grade II follicular non-Hodgkins lymphoma	DLBCL
Stage***	IIE	IIE	IIE	IIE
Prognostic index	Low-intermediate risk (IPI 2/5)	Low-intermediate risk (IPI 2/5)	Low risk (FLIPI 1/5)	Low-intermediate risk (IPI 2/5)
Treatment	R-CHOP * 4 then involved-field RT	CHOP * 3 then involved-field RT	Involved-field RT then CVP then rituximab	3 * CHOP then involved-field RT
Outcome	NED at 23 months	Local recurrence at 32 months post-diagnosis, with poor tolerance of 2^nd ^line chemotherapy and rapid treatment-related death	NED at 25 months	Out of field recurrence at 21 months post diagnosis. Salvage chemotherapy and autologous SCT. NED at 64 months from diagnosis

CHOP – Cyclophosphamide, doxorubicin, vincristine, prednisone

CVP – Cyclophosphamide, vincristine, prednisone

DLBCL – diffuse large B-cell non-Hodgkin's lymphoma

FLIPI – Follicular lymphoma international prognostic index [8]

IPI – International Prognostic Index for the Aggressive Non-Hodgkin's Lymphomas [7]

NED – No evidence of disease

R-CHOP – Rituximab, cyclophosphamide, doxorubicin, vincristine, prednisone

RT – Radiotherapy

SCT – Stem cell transplantation

* Normal Range 0–50 kU/L

** Histology defined according to REAL classification.[5]

*** Staging defined according to Ann Arbor staging system.[4]

**Table 2:** Literature review – patient characteristics, treatment and outcomes – abbreviations and reference list

**Name (Country)**	**Year**	**n**	**Pathology (number of cases)**	**Successful non-operative biopsy**	**Resection**	**Chemotherapy**	**Radiotherapy**	**Outcome without resection, chemotherapy or RT**	**Outcomes with resection**	**Outcomes with chemotherapy without resection or RT**	**Outcomes with chemotherapy and RT without resection**
**Hart USA**	1987	14	Histiocytcic (9) Poorly diff lymphocytic (2) Well diff lymphocytic (1) T cell (1) Undifferentiated (1)	0 of 14	1 of 14	CHOP (14)	1 of 14	0	1 NR (Received CHOP, RT NR)	6 alive at mean 32 months (18–48 months) 2 died within 3 months 5 died at mean 35 months (6–108) (5 of 13 received RT)	
**Webb USA**	1989	2**	DLBCL (6) Follicular mixed (2) Small cleaved (1)		1 of 2	MACOP-B (1)	0 of 2	0	1 alive at 95 months (Received CAMEL, 0 RT)	1 alive at 21 months	0
**Mansour England**	1989	12	NR	0 of 12	1 of 12	CHOP (9)	5 of 12	2 died in post-op period after bypass	1 NR (Received chemo, RT NR)	4 alive at 3,36,48,84 months 5 died at 2–11 months (5 of 9 received RT)	
**Tuchek USA**	1993	7	NR	0 of 7	0 of 7	CHOP (1) CVP (1) CAMEL (1)	0 of 7	4 died – mean survival 5 months	0	3 alive at 60,72,96 months	0
**Behrns USA**	1994	12	DLBCL (7) Small cleaved (2) Mixed (3)	4 of 12	1 of 12	CHOP (4)	4 of 12	2 relapsed or died (NR)	1 NR (Received chemo, RT NR)	2 died – mean survival 13 months	3 died – mean survival 26 months (5 died after RT only – mean survival 22 months)
**Ezzat Saudi Arabia**	1996	5	DLBCL (5)	2 of 5	1 of 5	CHOP (4) CHOP- Bleo (1)	2 of 5	0	1 alive at 23 months (Received CHOP, 0 RT)	2 alive at 24,24 months	2 alive at 30,84 months
**Bouvet USA**	1997	11	DLBCL (10) Mixed follicular (1)	6 of 11	3 of 11	See legend*	7 of 11	0	3 alive at 11,13,23 months (All received CHOP, OPEN or CHOP-Bleo, 0 RT)	1 alive at 191 months	4 alive at 44,55,67,159 months 3 died at 12,16,80 months
**Koniaris USA**	2000	8	DLBCL (7) Mixed follicular (1)	3 of 8	3 of 8	CHOP (6) MACOP-B (1)	5 of 8	0	3 alive at 53,62,64 months (2 received CHOP and RT)	1 alive at 24 months	2 alive at 51,128 months 2 died at 9,37 months
**Nishimur a Japan**	2001	19	DLBCL (9) B Immunocytoma (2) B follicular (2) T peripheral (3) T anaplastic large (1)	2 of 19	10 of 19	CHOP (5) Mitomycin (1) Tegafur (1) Unspecified chemo (1)	0 of 19	7 died at 1,2,2,3,8,60,72 months	4 died at 9,10,11,27 months 4 alive at 3,4,12,19 months 2 ND (6 received chemo, 4 no chemo, 0 RT)	2 died at 2,8 months	0
**Nayer USA**	2004	8	DLBCL (4) High grade B cell (1) Low grade B (2) Suspicious (1)	8 of 8	0 of 8	Chemo (4) Chemo and auto SCT (2) NR (2)	3 of 8 (2 NR)	0	0	3 alive at 5,6,76 months	3 alive at 2,7,72 months
**Arcari Italy**	2005	5	DLBCL (3) Lymphoplasmacytic (2)	3 of 5	2 of 5	CVP/CHO P (3) CHOP (1) CVP (1)	2 of 5	0	1 alive at 160 months 1 died at 8 months (Received CHOP or CVP, 0 RT)	1 alive at 60 months 2 died at 67, 88 months (2 of 3 received RT)	
**Grimison Australia [current series]**	2005	4	DLBCL (3) Follicular (1)	1 of 4	0 of 4	CHOP (2) Rituximab- CHOP (1) CVP (1)	4 of 4	0	0	0	1 died 32 months 3 alive 15,25,64 months
**Overall**		107 cases inc. current series		Excluding 2 NR: 29 of 105	23 of 107	80 of 107	Excluding 2 NR: 33 of 105	Excluding 2 NR: 0% (0 of 15) NED Mean 13 months 2YS 13% (2 of 15)	Excluding 5 NR: 72% (13 of 18) NED Mean 34 months 2YS 33% (6 of 18)	57% (36 of 63) NED Mean 38 months 2YS of 62% (28 of 45) – excludes 18 NR	

*CHOP (1), CHOP-Bleomycin (2), CHOP-OPEN (2), CHOP-Bleomycin, CMED, ESHAP (1), CHOP-Bleomycin, OPEN (1), CHOP, OAP-Bleomycin, MIME (1), CHOP-Bleomycin, MINE, ESHAP, Autologous SCT (1), IM-Etop, HOP (1), ESHAP, BACOS, MIME (1)

**excludes 7 patients with stage III/IV NHL

BACOS – bleomycin, doxorubicin, cyclophosphamide, vincristine, methylprednisolone

CAMEL – cyclophophamide, adriamycin, vincristine, prednisone Chemo – chemotherapy

CHOP – cyclophosphamide, doxorubicin, vincristine, prednisone

CMED – cyclophosphamide, methotrexate, etoposide, dexamethasone

CVP – cyclophosphamide, vincristine, prednisone

DLBCL – diffuse large Bcell lymphoma

ESHAP – etoposide, methyprednisolone, high-dose cytarabine, cisplatin

HOP – doxorubicin, vincristine, prednisone

IM-etop – ifosfamide, methotrexate, etoposide

MACOP-B

MIME – MESNA, ifosfamide, methotrexate, etoposide

MINE – MESNA, ifosfamide, mitoxantrone, etoposide

NED – no evidence of disease

NR – not reported

OAP-Bleomycin – vincristine, cytarabine, prednisone, bleomycin

OPEN – vincristine, prednisone, etoposide, mitoxantrone

RT – radiotherapy

SCT – stem cell transplantation

References for table 2 – Hart[12], Webb[22], Mansour.[45], Tuchek[47], Behrns.[2], Ezzat[48], Bouvet.[21], Koniaris[23], Nishimura[49], Nayer.[3], Arcari[20]
